# Family Commitment, Appreciation, and the Pursuit of Future Goals Among Young Adult Caregivers

**DOI:** 10.1093/geroni/igaf122.2976

**Published:** 2025-12-31

**Authors:** Maya Brown, Sibo Gao, Karen Fingerman

**Affiliations:** The University of Texas at Austin, Austin, Texas, United States; University of Texas at Austin, Austin, Texas, United States; The University of Texas at Austin, Austin, Texas, United States

## Abstract

Up to 10 million young adults are involved in caregiving for older family members with physical or cognitive impairments, but it is not clear whether these young adults feel acknowledged for their efforts. Family commitment may foster feelings of appreciation within the caregiving network. However, this sense of commitment and feeling appreciated could hinder the pursuit of future goals (e.g., education, romantic relationships) due to their greater investment in providing care. African American, Hispanic, Asian American and non-Hispanic White caregivers aged 18 to 29 (N = 141) of family members aged 65+ completed a 30-minute survey regarding caregiving tasks, family commitment, feelings of appreciation from other members of the caregiving network, ability to pursue future goals, and background information. Linear regression models revealed that young adult caregivers with higher family commitment reported greater feelings of appreciation from others in their caregiving network, controlling for caregiving tasks. Contrary to the hypothesis, young adult caregivers reported a greater ability to pursue future goals when they had higher family commitment and felt more appreciated. Findings suggest that young adult caregivers may feel more appreciated when their values (i.e., commitment) are consistent with their behaviors (i.e., providing care for older family member). This sense of appreciation may enhance their pursuit of future goals, likely because they integrate caregiving into their identity rather than viewing it as nonnormative. These issues may be more specific to young adults than midlife and older caregivers and warrant distinct interventions.

